# The impact of fatty acid synthase on HSV-1 infection dynamics

**DOI:** 10.1371/journal.ppat.1013068

**Published:** 2025-05-06

**Authors:** Camilla Albano, Linda Trifirò, Weronika Hewelt-Belka, Dana M. Cairns, Selina Pasquero, Gloria Griffante, Francesca Gugliesi, Greta Bajetto, Dorota Garwolińska, Marika Rossi, Marta Vallino, Mario Malerba, Marco De Andrea, David L. Kaplan, Valentina Dell’Oste, Matteo Biolatti

**Affiliations:** 1 Department of Public Health and Pediatric Sciences, University of Turin, Turin, Italy; 2 Department of Analytical Chemistry, Gdańsk University of Technology, Gdańsk, Poland; 3 Department of Biomedical Engineering, Tufts University, Medford, Massachusetts, United States of America; 4 CAAD Center for Translational Research on Autoimmune and Allergic Disease, University of Piemonte Orientale, Novara, Italy; 5 Institute for Sustainable Plant Protection, National Research Council of Italy, Turin, Italy; 6 INRIM Istituto Nazionale di Ricerca Metrologica, Turin, Italy; University of Wisconsin-Madison, UNITED STATES OF AMERICA

## Abstract

Herpes simplex virus type-1 (HSV-1) is a widespread human pathogen that relies on host cell pathways, including those involved in metabolism to support replication. Here, we demonstrate that *de novo* lipogenesis is essential for HSV-1 infectivity. Specifically, HSV-1 infection upregulates fatty acid synthase (FASN) expression, accompanied by a marked increase in lipids and a differential lipid species distribution. Conversely, silencing FASN or applying FASN inhibitors (*i.e*., CMS121 and C75) markedly reduces the infectivity of newly released HSV-1 virions, suggesting that, while initial replication remains unaffected, FASN is crucial for maintaining virion structure and facilitating entry into host cells. Additionally, we show that a source of lipid-rich external factors provided by fetal bovine serum significantly increases HSV-1 infectivity. Specifically, HSV-1 infection enhanced CD36-mediated fatty acid uptake, especially in FASN-depleted cells, compensating for reduced lipogenesis. Blocking CD36 function with SSO further decreased viral infectivity, demonstrating the critical role of lipid uptake in HSV-1 life cycle. Altogether, our findings reveal how HSV-1 manipulates lipid metabolism, offering insights into its association with chronic disease and therapeutic intervention.

## Author summary

Herpes simplex virus type-1 (HSV-1) manipulates host cell metabolism to support its replication. In this study, we highlight the essential role of *de novo* lipogenesis in HSV-1 infectivity. Infection with HSV-1 increases fatty acid synthase (FASN) expression, resulting in increased lipid production and altered lipid distribution. Disrupting FASN through silencing or inhibitors, like CMS121 and C75, diminishes viral infectivity by impairing virion structure and entry into host cells. Indeed, external lipid sources boost HSV-1 infectivity by enhancing CD36-mediated fatty acid uptake, particularly in FASN-depleted cells, compensating for reduced lipogenesis. Our findings provide significant insights into HSV-1 exploitation of lipid metabolism and its potential connection to chronic disease, highlighting new avenues for therapeutic intervention.

## Introduction

Herpes simplex virus type-1 (HSV-1) is a neurotropic double-stranded DNA virus primarily infecting the mucosal epithelium of the mouth, nose, or eyes [1]. Upon infection, HSV-1 establishes lytic infection and undergoes multiple rounds of viral replication in epithelial cells. Newly produced viral particles can then enter sensory neurons and travel via retrograde axonal transport to the trigeminal ganglion, where they release viral DNA and establish latent infection. Reactivation of the virus leads to the anterograde transportation of newly formed viral particles back to the initial infection site along sensory neurons, resulting in clinical lesions known as cold sores and fever blisters.

Emerging studies have unveiled an interesting interplay between herpesvirus infection and cellular lipid metabolism. Indeed, lipid metabolism, crucial for a range of cellular functions, appears to orchestrate key stages of the viral life cycle, ranging from entry and replication to the assembly of new virion particles. Thus, understanding how lipid metabolism is altered after viral infection may reveal potential targets for viral inhibition.

Extensive research has explored the interaction between human cytomegalovirus (HCMV) [2,3], a major human pathogenic β-herpesvirus, and host lipid metabolism due to well-known host cell stimulation by this infection. In contrast, the link between α-herpesvirus infection and lipids, such as during HSV-1 infection, known for its host shut-off properties [4], remains poorly understood [5–8]. Previous studies suggest that HSV-1 manipulates host lipid metabolism. For instance, Langeland *et al.* were the first to report that HSV-1 infection alters the synthesis of phosphoinositides, crucial signaling lipids [5]. Furthermore, it is known that phospholipids are synthesized anew for HSV-1 viral envelope formation [6,7]. Conversely, while HCMV relies on *de novo* fatty acid (FA) biosynthesis to produce mature enveloped particles, considerable evidence has shown that HSV-1 makes use of pre-existing membranes, favoring rapid nucleotide biosynthesis essential for a relatively short replicative cycle [8]. However, these findings do not fully explain how HSV-1 interacts with lipid metabolic pathways to enhance its replication and infectivity, leaving a critical knowledge gap.

To address this, we hypothesize that HSV-1 actively modulates *de novo* lipogenesis, particularly through the fatty acid synthase (FASN), a key enzyme in lipogenesis, to support viral maturation and infectivity. This hypothesis is supported by observations that Kaposi’s sarcoma-associated herpesvirus (KSHV), another herpesvirus, stimulates fatty acid synthesis to sustain its life cycle, with inhibitors of this pathway showing therapeutic promise [9]. Moreover, metabolic reprogramming is a common strategy among viruses, as seen in hepatitis C virus (HCV) and rhinovirus, which modulate glucose and lipid metabolism to optimize their replication [10,11]. Against this background, our findings reveal that HSV-1 significantly upregulates FASN activity across various cell types. This upregulation correlates with increased lipid concentrations and remodeling, suggesting a conserved mechanism by which HSV-1 manipulates lipid synthesis for virion assembly and infectivity. Our study further highlights the compensatory role of CD36-mediated fatty acid uptake in FASN-depleted cells, underscoring the dual reliance of HSV-1 on both endogenous lipid synthesis and exogenous lipid acquisition. Importantly, pharmacological inhibition of FASN not only disrupted lipid metabolism but also impaired virion integrity and infectivity, marking FASN as a critical host factor and potential therapeutic target.

In this paper, we aim to provide insights into the metabolic strategies employed by HSV-1 to sustain its replication cycle and, in the long term, their potential implications for counteracting chronic diseases caused by viral infections, like Alzheimer’s disease (AD). This rationale underpins the need for further research into lipid metabolism as a therapeutic target for combating both viral infections and the chronic diseases they may contribute to.

## Results

### HSV-1 infection modulates the fatty acid synthase

It has been confirmed that the fatty acid synthase (FASN), a key enzyme in the *de novo* lipid synthesis, regulates the replication of numerous viruses [12,13]. To investigate whether HSV-1 modulates FASN during infection, we conducted experiments using SH-SY5Y neuron-like cells, a suitable *in vitro* model for HSV-1-associated neurological disorders [14], under fully confluent and serum-free conditions, which allowed us to determine whether the observed lipid production is *de novo* rather than derived from external nutrient uptake.

**Fig 1:**
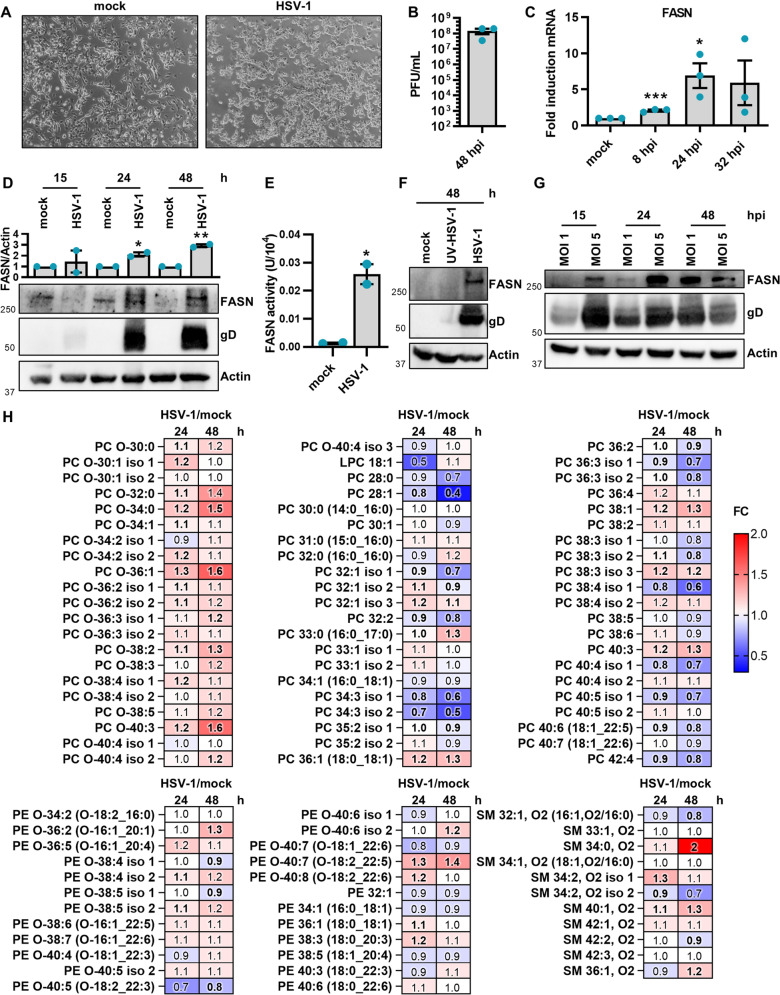
HSV-1 infection modulates the fatty acid synthase. SH-SY5Y cells were infected with HSV-1 (MOI 1) or left uninfected (mock) under serum-free conditions. **(A)** Representative images of mock- or HSV-1-infected cells at 48 h. **(B)** The extent of HSV-1 replication was assessed by titrating the infectivity of supernatants and cell-associated viruses combined using a standard plaque assay (*n* = 3). **(C)** At 8, 24, and 32 hours post-infection (hpi), total RNA was isolated and subjected to RT-qPCR to quantify FASN mRNA expression levels. The values were normalized against GAPDH mRNA and expressed as fold induction relative to mock-infected cells (set at 1) (*n* = 3; unpaired t-test). **(D)** Western blot analysis of protein lysates from mock or infected cells using antibodies against FASN, gD, or actin. A representative blot and densitometric analyses are shown. The values were normalized to actin and expressed as fold induction relative to mock-infected cells (set at 1). (*n* = 2; unpaired t-test). **(E)** The FASN activity was examined in mock- and HSV-1-infected SH-SY5Y cells (*n *= 2; unpaired t-test). Data are shown as the mean ± SEM, *P < 0.05, **P < 0.01, ***P < 0.001. **(F)** SH-SY5Y cells were infected with either HSV-1 or UV-inactivated HSV-1 (UV-HSV-1). Western blot analysis of protein lysates from mock or infected cells using antibodies against FASN, gD, or actin. A representative blot is shown (*n* = 2). **(G)** Western blot analysis using protein lysates from SH-SY5Y cells infected with different MOIs (1 and 5), probing for FASN, gD, or actin. A representative blot is shown (*n* = 2). **(H)** Heat map displaying the levels of glycerophosphocholines (PCs) and their ether analogs (PC-Os), glycerophosphoethanolamines (PEs) and their ether analogs (PE-Os), and sphingomyelins (SM) in HSV-1-infected cells compared to mock cells at 24 h (left column) and 48 h (right column). Upregulated (red, fold change > 1) and downregulated (blue, fold change < 1) lipid species are shown. Bold values indicate statistical significance (*P* < 0.05, unpaired t-test). The statistical evaluation for the lipid analysis of infected samples was performed using biological replicates (*n* = 2) for each condition as well as technical replicates (*n *= 2). For the control uninfected condition at the 24 hour time point one biological replicate was available. This extract was analyzed twice (technical replicates), and the results were averaged to produce additional representative values for the statistical analysis. For the 48 hour time point, two biological replicates were available for the control condition.

To validate the suitability of our experimental model, we first confirmed that serum-starved SH-SY5Y cells fully support virus replication (Fig 1A, B). Next, to test our hypothesis regarding the impact of HSV-1 on FASN, we quantified the FASN mRNA expression levels in SH-SY5Y cells previously infected with HSV-1 at 8, 24, and 32 hours post-infection (hpi). We found a significant increase in FASN mRNA in HSV-1-infected *vs.* uninfected cells, especially at 24 hpi, at which time point FASN levels were seven-fold higher than those in mock cells (Fig 1C). Subsequent Western blot analysis and activity assay provided further confirmation of FASN induction during the progression of infection, indicating an upregulation of lipogenesis (Fig 1D, E). These results were further validated in human foreskin fibroblasts (HFFs) and in HaCaT cells (S1 Fig). Furthermore, we observed that a UV-inactivated HSV-1 failed to induce FASN expression indicating that FASN upregulation requires active virus replication (Fig 1F). Lastly, as depicted in Fig 1G, the analysis of infection kinetics across various multiplicity of infections (MOIs) revealed that a higher MOI accelerates the upregulation of FASN protein expression. In particular, at an MOI of 5, we observed a significant increase in FASN protein levels at both 15 and 24 hpi in comparison with the levels seen with an MOI of 1. Similar results were obtained using other HSV-1 strains (*i.e.,* HSV-1 MacIntyre and 17+) emphasizing that this phenomenon is not strain-dependent (S2 Fig). Altogether, these findings support the involvement of HSV-1 in promoting the process of *de novo* lipogenesis following infection.

Next, to verify whether the differences in lipogenic enzyme expression between mock- and HSV-1-infected cells (MOI 1) led to changes in lipid composition, we performed lipidomic analysis by liquid chromatography coupled with high-resolution mass spectrometry (LC-Q-TOF-MS) at 24 and 48 h. These time points were chosen to capture a stage where cells were fully infected but still attached (Fig 1A), and FASN expression was upregulated (Fig 1C-E), ensuring that the virus late maturation phase, where FASN is anticipated to play a crucial role, was included. First, we compared total lipid amounts between infected and uninfected SH-SY5Y cells, using the total MS signal. This comparison included the following lipid classes: glycerophosphocholines (PCs) and ether analogs (PC-Os), glycerophosphoethanolamines (PEs) and ether analogs (PE-Os), sphingomyelins (SMs), and cholesterol. We observed an increase in the overall lipid content of PCs, PC-Os, PEs, PE-Os, SMs, and cholesterol in infected cells compared to their uninfected counterparts. For instance, at 24 h, the average fold change (FC) of total lipid amount in infected *vs.* noninfected cells was recorded as 1.28 for PCs, 1.41 for PC-Os, 1.46 for PEs, 1.42 for PE-Os, 1.38 for SMs, and 1.41 for cholesterol (S3 Fig).

Subsequently, we evaluated the differences in lipid composition within specific lipid classes in HSV-1 infected cells *vs.* uninfected SH-SY5Y cells. This comparison, addressing the percentage distribution of lipid species among SMs, PCs, and PEs, along with their ether analogs, revealed significant changes in cell lipid composition during infection, primarily within the PC class and its ether analogs (Fig 1H). Out of the 99 lipid species examined, 46 species at 24 h, and 38 at 48 h, showed significant changes between infected and uninfected cells (*P* < 0.05, unpaired t-test, false discovery rate - FDR). Almost all identified PC-Os were upregulated in HSV-1-infected cells compared to uninfected ones. Conversely, many long-chain (sum of carbon atoms in FAs ≥ 36) and unsaturated PCs (double bonds ≥ 3) were downregulated in HSV-1-infected cells, including PC 40:4, PC 40:5, PC 36:3, and PC 38:4. Unlike PCs, the percentage distribution of PEs and their ether analogs as well as SMs remained largely unchanged between HSV-1 infected and uninfected cells, with only a few exceptions.

### FASN modulation impacts HSV-1 replication and infectivity

**Fig 2:**
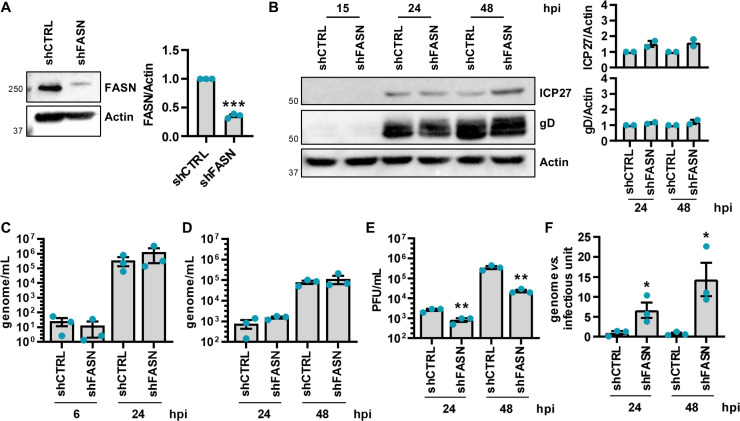
FASN modulation impacts HSV-1 replication and infectivity. SH-SY5Y cells were transduced with lentivirus delivering short hairpin RNA (shRNA) targeting FASN (shFASN) or scramble RNA control (shCTRL). **(A)** The efficiency of FASN protein depletion was assayed by immunoblotting using antibodies against human FASN or actin as a loading control. A representative blot (left) and the densitometric analyses (right) are reported (*n* = 3; unpaired t-test). **(B)** shCTRL and shFASN cells were infected with HSV-1 (MOI 1) under serum-free conditions. Western blot analysis of protein lysates from mock or infected cells at the indicated hpi using antibodies against ICP27, gD, or actin. A representative blot and the densitometric analyses are shown. Values were normalized to actin and plotted as fold induction relative to mock-infected cells (set at 1) (*n* = 2; unpaired t-test). **(C)** At 6 and 24 hpi, the number of intracellular HSV-1 genomes was measured by qPCR (*n* = 3; unpaired t-test). **(D)** At 24 and 48 hpi, the number of genome-containing particles released by cells was measured by qPCR (*n* = 3; unpaired t-test). **(E)** The supernatants from panel **D** were used to determine the number of infectious units/mL by the viral yield test (*n* = 3; unpaired t-test). **(F)** The genome-to-infectious unit ratio has been determined. Data are expressed as fold induction relative to shCTRL cells (set at 1) (*n* = 3; unpaired t-test). Data are shown as the mean ± SEM, *P < 0.05, **P < 0.01, ***P < 0.001.

To investigate the impact of FASN on HSV-1 infection, and since the potential role of lipids in ensuring the infectivity of viral particles, we next focused on the interplay between lipid metabolism and HSV-1 replication. For this purpose, SH-SY5Y cells were transduced with previously characterized lentiviral vectors delivering either a short hairpin RNA (shRNA) targeting FASN (shFASN) or a scrambled control RNA (shCTRL) under serum-free conditions. Transduced cells exhibited reduced FASN protein levels, confirming effective knockdown (KD) of FASN (Fig 2A), without affecting cell viability (S4 Fig). Following infection with HSV-1 (MOI 1), FASN depletion had no impact on the expression of HSV-1 ICP27 and gD proteins or on intracellular and extracellular viral loads (Fig 2B-D, respectively). However, FASN silencing significantly decreased both viral progeny production by >1-log (Fig 2E) and the infectivity of newly released HSV-1 virions (Fig 2F), suggesting that HSV-1 exploits FASN for virus production after the synthesis of late proteins, possibly during virion assembly.

### FASN inhibition increases fatty acid uptake and alters CD36 expression in HSV-1-infected cells

**Fig 3:**
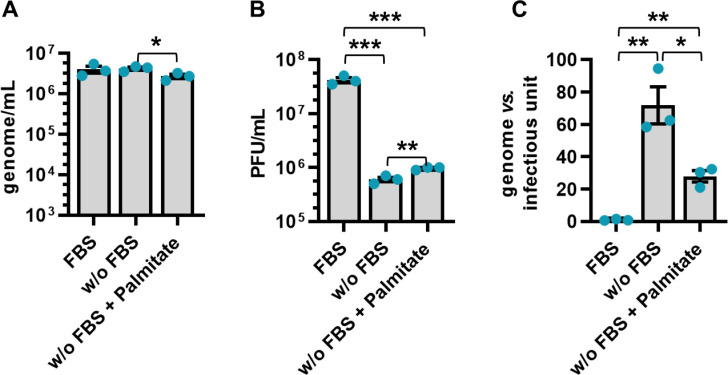
Palmitate rescues HSV-1 infectivity. SH-SY5Y cells were starved for 24 h and subsequently infected with HSV-1 (MOI 1). At 2 hpi the viral inoculum was removed, and cells were maintained under three different conditions: in the presence of serum, without (w/o) serum and without serum supplemented with palmitate (200 µM). **(A)** At 48 hpi, the number of genome-containing particles released by cells was measured by qPCR (*n* = 3; unpaired t-test). **(B)** The extent of HSV-1 replication was assessed by titrating the infectivity of supernatants using standard plaque assay (*n* = 3; unpaired t-test). **(C)** The genome-to-infectious unit ratio was calculated. Data are expressed as fold induction relative to FBS condition (set at 1) (*n* = 3; unpaired t-test). Data are shown as the mean ± SEM, *P < 0.05, **P < 0.01, ***P < 0.001.

Given the critical role of lipid metabolism in the life cycle of HSV-1 and the potential impact of lipid-rich external factors on this process, we explored the contribution of fetal bovine serum (FBS)—a standard ingredient in cell culture media essential for cell growth and maintenance—in HSV-1 replication. Although FBS is essential for cell culture, its role in virus research is complex due to its known effects on virus replication [15,16]. To further explore these phenomena, and verify the contribution of lipids in viral infectivity, we synchronized cells by serum starvation for 24 h and subsequently evaluated viral load, plaque formation, and infectivity under three conditions: cells cultured with FBS, without FBS, and without FBS but supplemented with palmitate (C16:0; a primary product of FASN activity). Our results showed that lipid availability—whether provided by FBS or palmitate supplementation—significantly enhanced viral particle infectivity (Fig 3). Notably, while palmitate supplementation in serum-free conditions partially restored infectivity, it did not fully recapitulate the effects observed in FBS-containing conditions. These findings highlight the critical role of lipids, potentially mediated by uptake mechanisms, in HSV-1 infectivity.

**Fig 4:**
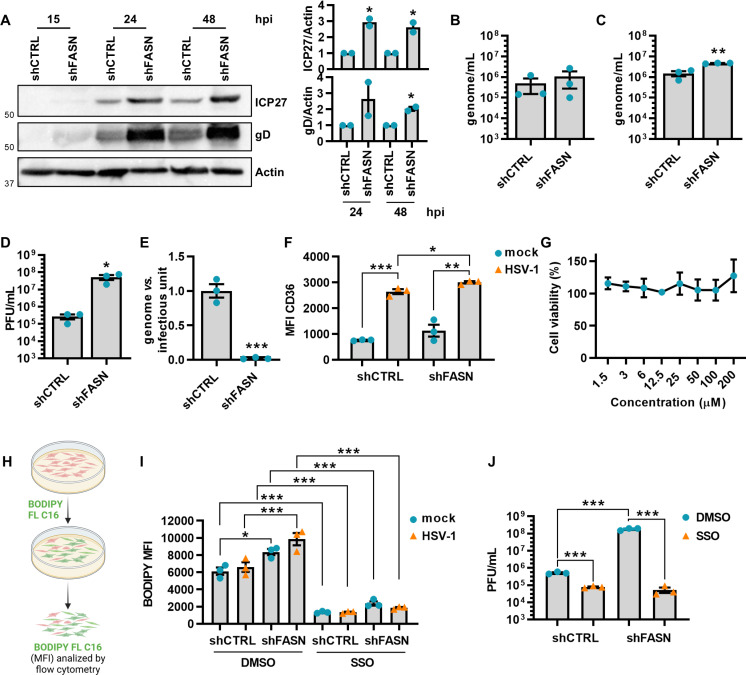
Effect of FASN knockdown on HSV-1 replication in serum conditions. shCTRL and shFASN cells were infected with HSV-1 (MOI 1). **(A)** Western blot analysis of protein lysates from mock or infected cells at the indicated hpi using antibodies against ICP27, gD, or actin. A representative blot and the densitometric analyses are shown. Values were normalized to actin and plotted as fold induction relative to mock-infected cells (set at 1). (*n* = 2; unpaired t-test). **(B)** At 24 hpi, the number of intracellular HSV-1 genomes was measured by qPCR (*n* = 3; unpaired t-test). **(C)** At 48 hpi, the number of genome-containing particles released by cells was measured by qPCR (*n* = 3; unpaired t-test). **(D)** The supernatants from panel **C** were used to determine the number of infectious units/mL by the viral yield test (*n* = 3; unpaired t-test). **(E)** The genome-to-infectious unit ratio has been determined. Data are expressed as fold induction relative to shCTRL cells (set at 1) (*n* = 3; unpaired t-test). **(F)** Surface CD36 protein expression in shCTRL and shFASN cells was assessed by flow cytometry. Results show the mean fluorescence intensity (MFI) (*n* = 3; unpaired t-test). **(G)** SSO toxicity was determined at 48 h post-treatment (hpt), using the MTT method (*n* = 3). **(H)** Schematic representation of FA uptake using a fluorescent analog of palmitate (BODIPY FL C16) (Created in BioRender. De Andrea, M. (2025) https://BioRender.com/j23i568). **(I)** shCTRL and shFASN cells were infected with HSV-1 in serum-free conditions and at 48 hpi treated with DMSO or SSO (200 µM) for 1 h. Then, cells were cultured in the presence of 2 μM BODIPY FL C16 for 30 minutes and then assessed by flow cytometry. Results show the MFI (*n* = 3; one-way ANOVA followed by Bonferroni’s post-tests). **(J)** shCTRL and shFASN cells were treated with DMSO or SSO in the presence of serum and then infected with HSV-1 (MOI 1). At 48 hpi, the supernatants were used to determine the number of infectious units/mL by the viral yield test (*n* = 3; unpaired t-test). Data are shown as the mean ± SEM, *P < 0.05, **P < 0.01, ***P < 0.001.

Next, to comprehensively assess the impact of FASN on HSV-1 expression, we conducted a set of experiments in the presence of serum in our cellular model (S5 Fig). The analysis of viral protein expression (Fig 4A) revealed that FASN depletion considerably raised the expression levels of both ICP27 and gD in the presence of serum. Moreover, intracellular and extracellular viral genome copies, as well as cell infectivity, were also notably increased (Fig 4B-E). The aforementioned results led us to postulate that inhibiting FASN could trigger an increased uptake of FAs from the serum to compensate for the lack of this enzyme. To validate this hypothesis, we initially analyzed the expression of CD36, a key FA transporter [17]. Since CD36 acts at the cell surface, we used flow cytometry to determine its localization in SH-SY5Y cells following HSV-1 infection. Despite evidence showing widespread host shut-off during infection [18], we found that HSV-1 infection enhanced CD36 plasma membrane localization, especially in shFASN cells (Fig 4F). To determine whether CD36 increased localization correlated with enhanced FA transport activity, we measured exogenous FA uptake using a fluorescent analog of palmitate (BODIPY FL C16) (Fig 4H). In line with previous reports, flow cytometry analysis revealed enhanced uptake of the FA analog in HSV-1-infected cells compared to uninfected control cells at 48 hpi, with shFASN cells showing an even greater uptake of BODIPY (Fig 4I). Furthermore, to assess the inhibition of FA uptake via CD36, we employed sulfosuccinimidyl oleate (SSO), a compound that can inhibit CD36 function by binding to Lys164 in its hydrophobic cavity [19]. At a non-toxic concentration of 200 μM, as judged by MTT assay (Fig 4G), SSO markedly reduced CD36-mediated FA uptake in both HSV-1-infected shFASN and shCTRL cells. Collectively, these data indicate that HSV-1 infection enhanced plasma membrane localization of CD36 and FA uptake, especially in the absence of FASN.

Next, we sought to determine whether blocking FA uptake via CD36 would affect HSV-1 infectivity. For this purpose, both shCTRL and shFASN cells were treated with SSO or vehicle control (DMSO). In agreement with our previous results, we observed reduced infectivity of viral particles released in the supernatants of cells treated with SSO compared to those treated with DMSO, indicating a reduced capacity of the virus to form plaques (Fig 4J). Of note, the most pronounced difference in infectivity was detected between SSO- and DMSO-treated shFASN cells, suggesting that the combined effect of inhibiting lipogenesis and blocking CD36-mediated FA uptake reduced viral infectivity.

### CMS121 and C75 reduce the infectivity of HSV-1 viral particles

Given the capability of FASN to impair HSV-1 infectivity, we reasoned that targeting lipid metabolism may constitute an innovative approach to reduce the risk of virus reactivation, an especially critical concern in the context of neurodegenerative diseases, such as Alzheimer’s disease (AD). Given the increasing body of evidence linking HSV-1 to the onset of AD and the demonstrated neuroprotective effects of CMS121 — a fisetin-derived FASN inhibitor (FASNi) that reduces cognitive decline in mice [20,21] — we aimed to evaluate its impact on HSV-1 replication. In parallel, we also investigated the effects of C75, a synthetic α-methylene-γ-butyrolactone that targets *de novo* lipogenesis, ultimately aiming to establish a starting point for future investigations to provide a completely new approach to minimize chronic disease.

**Fig 5:**
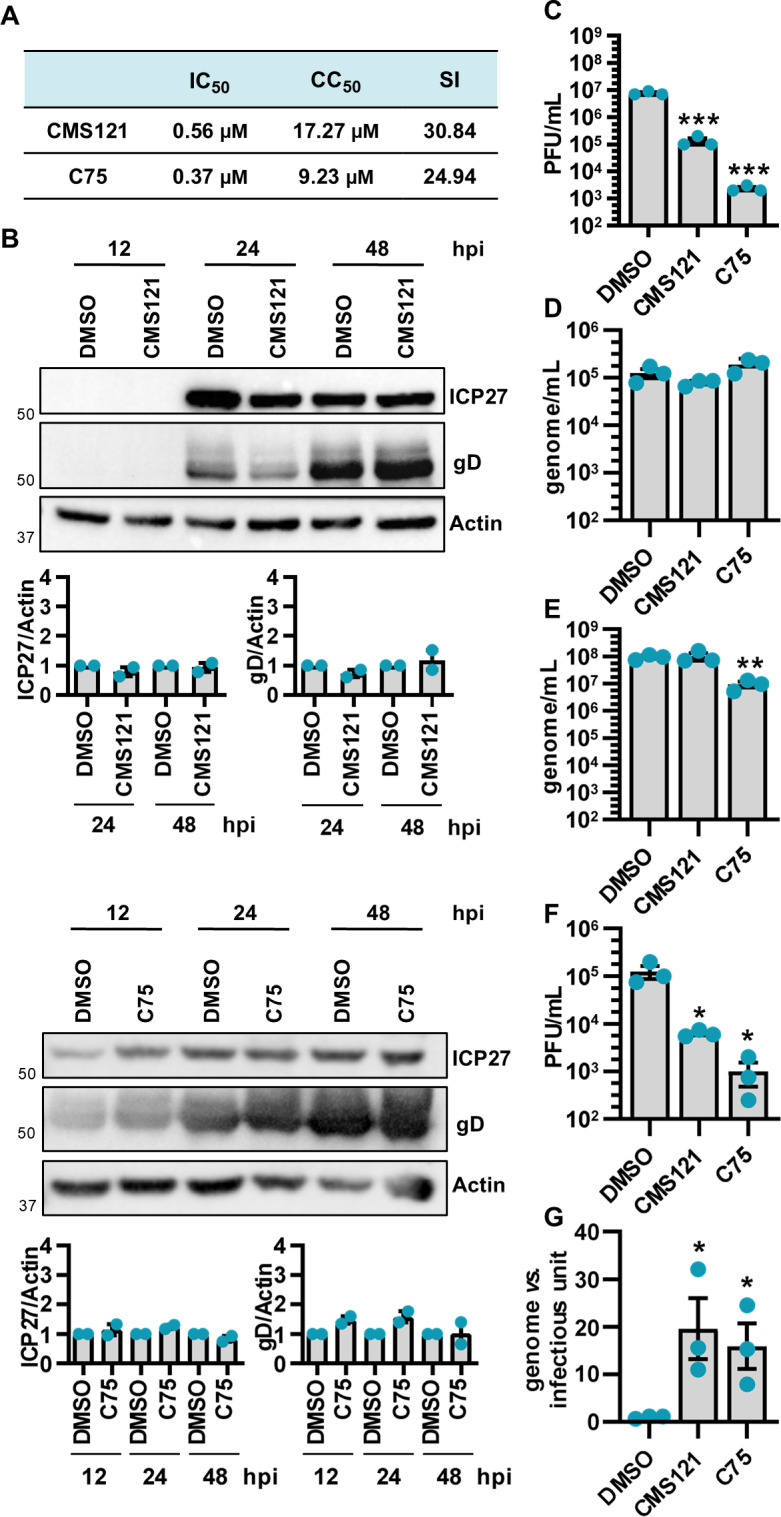
CMS121 and C75 reduce the infectivity of HSV-1 viral particles. **(A)** The CC_50_ (50% cytotoxic concentration), IC_50_ (50% inhibitory concentration), and selectivity index (SI) of CMS121 and C75 at 48 h in SH-SY5Y cells under serum-free conditions. **(B)** SH-SY5Y cells were infected with HSV-1 (MOI 1) and treated with CMS121, C75, or vehicle DMSO. Protein lysates from cells harvested at different time points were subjected to Western blot analysis using antibodies against ICP27, gD, or actin. A representative blot and the densitometric analyses are shown. Values were normalized to actin and plotted as fold induction relative to mock-infected cells (set at 1). (*n* = 2; unpaired t-test). **(C)** The extent of HSV-1 replication was assessed by titrating the infectivity of supernatants and cell-associated viruses from freeze-thaw cycles through standard plaque assay (*n* = 3; unpaired t-test). **(D)** At 24 hpi, the number of intracellular HSV-1 genomes was measured by qPCR (*n* = 3; unpaired t-test). **(E)** At 48 hpi, the number of genome-containing particles released by cells was measured by qPCR (*n* = 3; unpaired t-test). **(F)** Infectious units/mL were determined from supernatants in panel **E** by viral yield assay (*n* = 3; unpaired t-test). **(G)** The genome-to-infectious unit ratio was calculated as well. Data are expressed as fold induction relative to DMSO treated cells (set at 1) (*n* = 3; unpaired t-test). Data are shown as the mean ± SEM, *P < 0.05, **P < 0.01, ***P < 0.001.

We first determined the CC_50_ (50% cytotoxic concentration), IC_50_ (50% inhibitory concentration), and selectivity index (SI) of the CMS121 and C75 compounds (Fig 5A). Next, to investigate their antiviral activity, we adopted a similar approach previously utilized in shRNA experiments. Specifically, HSV-1-infected SH-SY5Y cells were treated with 5 µM CMS121 or C75, and viral protein expression was examined by immunoblotting. The expression of ICP27 and gD proteins remained unaffected upon both treatments (Fig 5B). However, standard plaque assays revealed a substantial decrease in the accumulation of infectious viral progeny in cells treated with CMS121 or C75 compared to untreated controls at 48 hpi (Fig 5C). Quantification of intracellular and extracellular HSV-1 genomes in infected cells (MOI 1) treated with CMS121 revealed no significant differences compared to vehicle-treated controls, indicating that viral replication was not affected. A similar trend was observed for C75 treatment, although a reduction in extracellular viral genomes was detected in C75-treated cells (Fig 5D, E). To determine viral infectivity post-treatment, we measured the number of infectious units/mL by viral yield assay. This revealed a considerable reduction in the infectivity of virions released by FASNis-treated cells compared to vehicle-treated ones, indicating a diminished ability of the virus to form plaques (Fig 5F). This result was further validated by the genome-to-infectious unit ratio, which confirmed a substantial decrease in the infectivity of virions released by treated cells compared to untreated ones (Fig 5G). These findings indicate that CMS121 and C75 exert their inhibitory effects at later stages of virion maturation, specifically affecting the quality of the virions produced as well as their ability to infect new cells.

**Fig 6:**
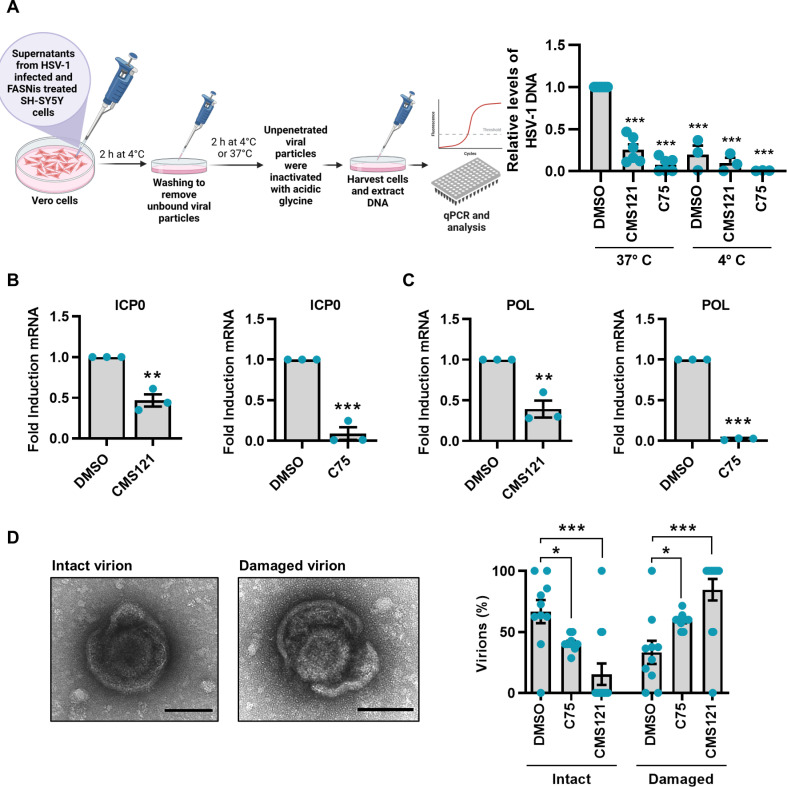
Impact of CMS121 and C75 on HSV-1 entry into Vero cells. **(A)** Schematic representation of entry assay (left panel; Created in BioRender. De Andrea, M. (2025) https://BioRender.com/b96q288). HSV-1 genomes present in infected cells were measured by qPCR using primers for the gE gene, and values were normalized to the housekeeping gene GAPDH (right panel). Data are expressed as fold induction relative to DMSO treated cells at 37° C (set at 1) (at least *n* = 3; unpaired t-test). At 6 hpi, total RNA was isolated and subjected to RT-qPCR to measure mRNA expression levels of HSV-1 ICP0 **(B)** and POL **(C).** Values were normalized to the housekeeping GAPDH and plotted as fold induction relative to DMSO-treated infected cells (set at 1) (*n* = 3; unpaired t-test). **(D)** Representative image of an intact HSV-1 virion showing typical herpesvirus morphology with a prominent outer envelope (left) or a damaged virion displaying compromised envelope integrity (right). Scale bars 100 nm. The number of enveloped particles was counted on different frames in DMSO-, C75- or CMS121-treated cells and plotted as a percentage (intact *vs.* damaged) (unpaired t-test). Data are shown as the mean ± SEM, *P < 0.05, **P < 0.01, ***P < 0.001.

To ascertain whether CMS121 and C75 treatment affects the entry capability of HSV-1 virions, we used supernatants from HSV-1-infected SH-SY5Y cells treated with each compound or DMSO for 48 h to infect Vero cells. Following a 2 h HSV-1 entry period, we quantified the viral genome content of these cells. As shown in Fig 6A, both treatments significantly hindered the ability of newly formed HSV-1 viral particles to enter the cells. This observation was further substantiated by the reduced mRNA expression of ICP0, an HSV-1 immediate-early gene, and viral DNA polymerase (POL) at 6 hpi in similarly treated cells, suggesting that CMS121 and C75 compromise virions integrity, thus impairing entry capability (Fig 6B, C). To confirm our hypothesis regarding viral envelope disruption, we examined HSV-1 virion morphology in post-treatment supernatants using transmission electron microscopy (TEM). The typical icosahedral structure of herpesviruses capsid was observed in all specimens; however, the surrounding external envelope was not well preserved in treated samples, where it appeared discontinuous with one or more interruptions or almost completely degraded (Fig 6D, and S6 Fig). Altogether, these findings indicate that pharmacological inhibition of FASN disrupts the stability of HSV-1 surface structure and virion morphology.

Finally, we compared the total lipid amounts, as measured by total MS signal, across different classes between infected cells treated with FASNis and vehicle DMSO. In cells treated with either CMS121 or C75 there was a notable reduction in total lipid content in comparison to merely infected cells, encompassing all lipid classes (S3 Fig). For instance, FC of total lipid amount in infected cells treated with CMS121 *vs.* DMSO-infected cells at 24 hpi was 0.75 for PCs, 0.79 for PC-Os, 0.56 for PEs, 0.54 for PE-Os, and 0.8 for SMs. These changes were statistically significant at 24 hpi (*P* < 0.05) and 48 hpi (*P* < 0.001) in C75- *vs.* DMSO-treated infected cells, and at 24 hpi (*P *< 0.005) in CMS121- *vs.* DMSO-treated infected cells. The significant changes at 48 hpi for CMS121-treated infected cells were limited to SMs, cholesterol, and PCs (*P* < 0.05).

**Fig 7:**
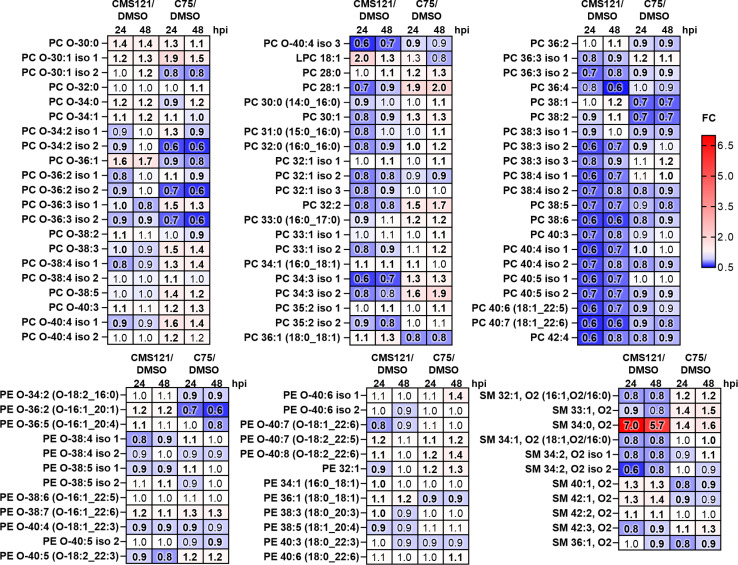
CMS121 and C75 modulate HSV-1-induced lipogenesis. Heat map of glycerophosphocholines (PCs), ether PC (PC-Os), glycerophosphoethanolamines (PEs), ether PE (PE-Os), and sphingomyelins (SMs) in SH-SY5Y cells infected with HSV-1 (MOI 1) and subsequently treated with CMS121, C75 or vehicle (DMSO) (24 hpi left column/48 hpi right column). Changes are represented for 24 and 48 hpi, with upregulation (red, fold change > 1) and downregulation (blue, fold change < 1) highlighted. Statistically significant alterations are denoted in bold (unpaired t-test, *P* < 0.05). Data are derived from at least two independent biological replicates analyzed in duplicates.

When comparing HSV-1 infected cells with CMS121-treated infected cells, 76 lipid species at 24 hpi, and 63 at 48 hpi, showed significant differences (Fig 7). In CMS121-treated cells, there was a noticeable decrease in the relative amounts of LC-PUFAs in PCs and some SMs, along with a slight increase in short-chained ether analogs of PCs and LPC 18:1. Conversely, in C75-treated cells, we observed an increase in the levels of several LC-PUFA ether analogs of PCs and PEs compared to DMSO-treated HSV-1 infected cells, whereas the relative amounts of LC-PUFAs in PCs were reduced. These changes were statistically significant for 63 lipid species at 24 hpi and 72 at 48 hpi (*P* < 0.05, unpaired t-test, FDR).

## Discussion

Viral infections are known to trigger metabolic changes in host cells to support the increased demand for nutrients, energy, and macromolecular synthesis. This phenomenon has been extensively studied in the context of HCMV, a prominent β-herpesvirus, and its interaction with host lipid metabolism [22–24]. However, the dynamics between α-herpesviruses, such as HSV-1, and lipid metabolism remain less clear, with existing literature often yielding conflicting evidence, likely ascribable to variations in growth conditions across studies that influence virus replication. For instance, an earlier study by Langeland *et al.* revealed alterations in phosphoinositide synthesis, a pivotal signaling lipid affected by HSV-1 [5], providing the first evidence of an interplay between this virus and host lipid metabolism. Subsequent studies demonstrated that phospholipids are synthesized anew in cells infected with HSV-1, serving as essential components for the formation of the viral envelope [6,7]. Recently, Arii *et al.* showed the importance of phosphatidylethanolamine biosynthesis in herpesvirus infections [25]. On the other hand, studies like that of Vastag *et al.* suggested that HSV-1 assembles its virions using pre-existing membranes to expedite nucleotide biosynthesis, essential for its rapid replicative cycle [8]. Given these contrasting findings, here we have further investigated the complex interplay between *de novo* lipid synthesis and HSV-1 infection in a neuronal-like context.

Our study provides new insights into the interplay between HSV-1 infection and lipid metabolism, particularly the role of fatty acid synthase (FASN) in promoting viral replication and infectivity. Our findings support the notion that HSV-1 directly modulates FASN expression to promote *de novo* lipogenesis during infection. We observed a significant increase in FASN mRNA and protein levels in different cell types, suggesting a conserved mechanism by which the virus exploits host lipid synthesis pathways. The subsequent confirmation of these results via enzymatic activity assays reinforces the conclusion that FASN is actively induced as part of the viral infection process. Crucially, our results indicate that FASN upregulation is strictly dependent on active viral replication, as demonstrated by the failure of UV-inactivated HSV-1 to induce FASN expression. This finding suggests that HSV-1 does not passively influence FASN levels through structural components or entry mechanisms but rather engages in an active regulatory process that requires viral gene expression and replication intermediates. Furthermore, the observation that FASN upregulation correlates with viral load and infection kinetics—where higher MOIs lead to accelerated FASN induction—further supports the hypothesis that HSV-1 actively exploits lipid biosynthesis to support its replication cycle.

This upregulation of FASN, coupled with increased lipid content and altered lipid composition, highlights the importance of *de novo* lipogenesis in the HSV-1 life cycle. The observed changes in lipid profiles, particularly the enrichment of glycerophospholipids such as PCs and PC-Os, emphasize the critical role of lipid remodeling in supporting HSV-1 virion assembly and infectivity. Notably, HSV-1 infection also led to the downregulation of long-chain polyunsaturated fatty acids (LC-PUFAs) in PCs, which could reflect specific adaptations in membrane composition to optimize viral replication and egress.

Functional studies further underscored the significance of FASN in HSV-1 pathogenesis. While FASN knockdown did not affect viral genome replication or protein expression, it substantially reduced the production of infectious progeny and impaired the infectivity of newly formed virions in serum-free conditions. Similar results were observed with pharmacological inhibitors of FASN (*i.e.,* CMS121 and C75). Both inhibitors dramatically reduced the infectivity of HSV-1 virions without affecting viral protein expression or genome replication, highlighting their impact on late-stage processes. Lipidomic analyses of inhibitor-treated cells revealed a significant decrease in total lipid content and altered lipid compositions, including reductions in LC-PUFAs and specific lipid species critical for maintaining membrane fluidity and integrity. Furthermore, transmission electron microscopy showed structural defects in HSV-1 virions produced under FASN-inhibitory conditions, indicating compromised envelope integrity. Together, these findings suggest that HSV-1 relies on FASN-mediated lipid synthesis during the late stages of its life cycle, likely influencing virion assembly and envelope integrity.

Moreover, our study reveals that lipid availability plays a crucial role in HSV-1 infectivity, as demonstrated by the observed differences in viral load, plaque formation, and infectivity across different culture conditions. When cells were serum-starved for 24 hours, viral infectivity was significantly reduced, indicating that the absence of extracellular lipids negatively impacts the virus ability to propagate efficiently. However, when palmitate (C16:0), a major product of FASN activity and a key component of lipid metabolism, was supplemented in serum-free conditions, infectivity was partially restored, although not to the same extent as in FBS-containing conditions. This partial restoration suggests that HSV-1 exploits host-derived fatty acids, such as palmitate, to facilitate its replication and infectivity. Since palmitate serves as a precursor for phospholipids and complex lipids essential for membrane integrity and viral envelope formation, its supplementation likely compensates for the lack of endogenous lipid synthesis during serum starvation. However, the fact that palmitate alone did not fully restore viral infectivity to FBS levels implies that additional lipid components present in serum, such as cholesterol, phospholipids, or other fatty acids, may also be required for optimal virion stability and host cell entry. Furthermore, these results suggest that HSV-1 can utilize both endogenous lipid synthesis via FASN and exogenous lipid uptake to support its life cycle. Supporting this, our results show that HSV-1 infection enhances the membrane localization of CD36, a key fatty acid transporter, particularly in FASN-depleted cells. This increased CD36 activity compensates for impaired lipogenesis by promoting exogenous fatty acid uptake, which in turn supports viral infectivity. Importantly, pharmacological inhibition of CD36 using SSO significantly reduced HSV-1 infectivity, particularly in FASN-depleted cells, indicating that both *de novo* lipogenesis and exogenous fatty acid uptake are critical for maintaining HSV-1 infectivity.

Together, these findings underscore the pivotal role of FASN as a host factor for HSV-1 replication and as a promising therapeutic target. However, future studies should focus on clarifying the precise mechanisms by which lipid remodeling supports HSV-1 assembly and infectivity, with a particular focus on defining the virion lipid composition. Additionally, investigations into the interplay between HSV-1 and host lipid metabolism in more complex *in vivo* models—especially those relevant to neurodegenerative diseases—will be crucial for advancing this field and assessing the translational potential of FASN inhibitors. Notably, previous studies have highlighted the potential of targeting lipid metabolism in Alzheimer’s disease (AD). Recently, Ates *et al.* [20] demonstrated that FASN protein expression is significantly elevated in AD patients, reinforcing the idea that targeting lipid metabolism could be beneficial. This is further supported by findings that CMS121 administration enhances memory and cognition in an AD mouse model, mirroring the protective effects of fisetin reported in earlier studies [21]. Beyond AD, the therapeutic relevance of FASN inhibition extends to various diseases, including cancer, fatty liver disease, obesity, and type 2 diabetes [26–29], underscoring its broad therapeutic potential.

In summary, our research advances the current understanding by demonstrating that the FASN inhibitors CMS121 and C75 effectively reduce HSV-1 viral infectivity. These findings underscore the potential of targeting lipid metabolism as a novel therapeutic strategy to mitigate the progression of AD, especially in the context of viral infections.

## Materials and methods

### Cells and viruses

SH-SY5Y cells (ATCC CLR-2266), African green monkey kidney cells (Vero; ATCC CCL-81), human foreskin fibroblasts (HFFs; ATCC SCRC-1041), HaCaT cells [30], and human embryo kidney 293 cells (HEK 293T; ATCC CRL-3216) were maintained at 37°C with 5% CO_2_. Cells were cultured in Dulbecco’s modified Eagle medium (DMEM), supplemented with 10% heat-inactivated fetal bovine serum (FBS), 2 mM glutamine, 1 mM sodium pyruvate, 100 U/mL penicillin, and 100 µg/mL streptomycin sulfate (Sigma-Aldrich). HEK 293T and SH-SY5Y cells were supplemented with 1% non-essential amino acids (Sigma-Aldrich).

The HSV-1 clinical isolate was a generous gift from Valeria Ghisetti (Amedeo di Savoia Hospital, Turin, Italy), the HSV-1 17+ was provided by Beate Sodeik (Hannover Medical School, Hannover, Germany), while the HSV-1 MacIntyre was purchased by ATCC (VR-539). They were propagated and titrated by plaque assay on Vero cells, as previously described [31].

For experiments conducted without serum, cells were initially cultured in a growth medium supplemented with serum. Twenty-four hours prior to infection, cells were subjected to serum starvation and subsequently maintained in serum-free conditions throughout the infection period. Infections were carried out using a MOI of either 1 or 5 infectious units per cell, tailored to the specific requirements of each experiment.

The particle-to-infectious unit ratio was determined by comparing the amount of viral DNA to the infectious virus yield in cell-free supernatants. The determination of virus yields from cell-free supernatants is described elsewhere [32]. After digestion with DNase I (Sigma-Aldrich), used to remove free nucleic acids and quantify only viral genome particles, viral DNA in supernatants was quantified by quantitative PCR (qPCR), using primers to amplify gE gene (S1 Table). Intracellular HSV-1 DNA copy numbers were normalized to glyceraldehyde 3-phosphate dehydrogenase (GAPDH) (S1 Table). A standard curve of serially diluted genomic DNA mixed with a gE-encoding plasmid was created in parallel with each analysis.

UV-inactivated HSV-1 was prepared using a double pulse of UV-B light (1.2 J/cm^2^). The UV-inactivated HSV-1 did not replicate or produce detectable levels of viral proteins.

### Reagents

CMS121 (HY-135981, MedChemExpress), C75 (HY-12364, MedChemExpress), SSO (11211, Cayman Chemical), palmitate (P0500; Sigma-Aldrich), and BODIPY FL C16 (D3821; Invitrogen) were used at a concentration of 5 μM, 5 μM, 200 μM, 200 μM, and 2 μM, respectively.

pLKO.1 puro-humanU6-shRNA FASN was a gift from Elizabeth Stoll (Addgene plasmid #82327; http://n2t.net/addgene:82327; RRID: Addgene_82327). pLKO.1 Puro shRNA Scramble was a gift from Antonia Follenzi (University of Piemonte Orientale, Novara, Italy). pAcUW51-CgE was a gift from Pamela Bjorkman (Addgene plasmid #13762; http://n2t.net/addgene:13762; RRID: Addgene_13762) [33].

### Lentivirus production

Recombinant lentiviruses were packaged in HEK 293T cells by cotransfection of the 3^rd^ Generation Packaging System Mix (kindly provided by Antonia Follenzi, University of Piemonte Orientale, Novara, Italy) with the above-mentioned vectors to produce viral particles using Lipofectamine 2000 (Thermo Fisher Scientific). Viral supernatants were harvested after 72 h and used to transduce SH-SY5Y cells by infection in the presence of 10 μg/mL polybrene. Transduced cells were selected with puromycin (1 μg/mL) over the course of 14 days post-transduction. After selection, the successful knockout was confirmed by immunoblotting.

### Cytotoxicity assay

To determine the cytotoxicity of CMS121, C75, and SSO, SH-SY5Y cells were seeded in a 96-well culture plate and exposed to increasing concentrations of either compounds or vehicle dimethyl sulfoxide (DMSO, Sigma-Aldrich) the following day. After 48 h of incubation, the number of viable cells was determined using the 3-(4,5-dimethylthiazol-2-yl)-2,5-diphenyltetrazolium bromide (MTT) (Sigma-Aldrich) assay, as previously described [34]. Similarly, shCTRL and shFASN cells were grown to confluence. The medium was then replaced with serum-free medium, and after 48 hours, the number of viable cells was measured using the MTT assay.

Moreover, crystal violet was used to assay cell viability in shCTRL and shFASN cells. Briefly, cells were grown to confluence, then the medium was removed, and serum-free medium was added. Cells were maintained in a serum-free medium for 48 hours. Cells were fixed and stained with a solution of 0.1% crystal violet and 20% ethanol in water for 20 min, after which the cells were washed three times with water and allowed to dry. Crystal violet dye was then solubilized by adding 10% acetic acid to each well and incubated for 20 min with shaking. Samples were quantified by the absorbance at 595 nm using a Victor X3 Multilabel Plate Reader (Perkin Elmer; Wallac 1420 workstation software).

### Viral entry assay

Vero cells were infected with the same amount of HSV-1 virions released by SH-SY5Y cells upon treatment with CMS121, C75, or DMSO for 48 h. HSV-1 was adsorbed for 2 h at 4 °C on pre-chilled cells to allow viral attachment. Cells were then washed with cold medium three times to remove the unbound virus and incubated for 2 h at 37 °C for virus entry or kept at 4 °C for 2 hours as a negative control. The unpenetrated virus was inactivated with acidic glycine for 2 min at room temperature. Cells were then washed with a warm medium three times to return the pH to neutral. Total DNA was extracted using TRI Reagent solution (Life Technologies) according to the manufacturer’s instructions. Finally, cell-penetrated HSV-1 genome copy numbers were measured by qPCR using primers for the gE gene, and values were normalized to the housekeeping gene GAPDH. The primers used are reported in S1 Table.

### RNA isolation and RT-qPCR

Total RNA was extracted using TRI Reagent solution (Life Technologies) according to the manufacturer’s instructions, and 1 μg was retrotranscribed using the Revert-Aid H-Minus FirstStrand cDNA synthesis kit (Thermo Fisher Scientific). Comparison of expression between samples was performed by SYBR green-based reverse transcription-qPCR (RT-qPCR) using a CFX96 Real-Time System apparatus (Bio-Rad Laboratories). The housekeeping gene GAPDH was used to normalize for variation in cDNA levels. The primers used are reported in S1 Table.

### Protein analysis

Whole-cell protein extracts were examined by Western blot analysis as previously described [35]. Brieﬂy, cells were lysed in ice-cold RIPA buffer supplemented with protease inhibitors (Sigma-Aldrich) to obtain total cell lysate. Equal amounts of cell extracts were fractionated by electrophoresis on SDS-polyacrylamide gels and transferred to Immobilon-P membranes (Merck Millipore). After blocking with TBST (Tris-buffered saline containing 0.05% Tween20) containing 5% milk, membranes were incubated overnight at 4°C with the appropriate primary antibodies. Membranes were then washed with TBST and incubated for 2 h at room temperature with HRP-conjugated anti-mouse or anti-rabbit secondary antibodies. Proteins were visualized using the ChemiDoc MP Imaging System (Bio-Rad Laboratories) and an enhanced chemiluminescence detection kit (Thermo Fisher Scientiﬁc). Scanning densitometry of the bands was performed using Image Lab (version 6.0.1; Bio-Rad Laboratories). The following primary and secondary antibodies were used: anti-actin clone C4 (MAB1501; Sigma-Aldrich); anti-FASN (3189S; Cell Signaling Technology), anti-HSV gD clone 2C10 (HA025; Virusys Corporation), anti-HSV ICP27 clone H1113 (P1113; Virusys Corporation), anti-mouse IgG (H+L)-peroxidase and anti-rabbit IgG (H+L)-peroxidase antibodies (3701095 and SAB3700934; Sigma-Aldrich).

### FACS CD36

Cells were seeded at a density of 4 × 10^5^ cells per well in a 12-well plate. Cells were harvested by PBS-EDTA 2mM and stained with CD36 primary antibody (PA1–16813; Invitrogen) combined with Alexa Flour 488 anti-rabbit (A11008; Life Technologies) mix solution for 1 h at 37°C. Cells were washed twice in PBS, fixed in PBS containing 1% paraformaldehyde (PAF), and analyzed on a BD FACSCanto II flow cytometer (BD Biosciences). Data were processed and analyzed using FlowJo software (BD Biosciences).

### BODIPY-C16 uptake assay

shCTRL and shFASN cells were seeded at a density of 4 × 10^5^ cells/well in a 12-well plate. Cells were infected with HSV-1 (MOI 1) in serum-free media. After 48 h, cells were treated with SSO for 1 h or left untreated and then cultured in serum-free media supplemented with 2 μM BODIPY FL C16 (D3821; Invitrogen) for 30 min. Subsequently, cells were harvested by trypsinization, washed twice with PBS, and fixed with PBS-PAF 1% for 20 min at room temperature and finally analyzed by flow cytometry using a BD FACSCanto II flow cytometer (BD Biosciences). Data were processed and analyzed using FlowJo software (BD Biosciences). The mean fluorescence intensity (MFI) was compared between samples.

### Transmission electron microscopy

HSV-1 virions were allowed to adsorb onto carbon and Formvar-coated copper grids (Pelco), left to stand for 5 min, washed with water, and then negatively stained using 0.5% aqueous uranyl acetate. Images were captured using a CM10 electron microscope (Philips) operated at 60 kV. Part of the analysis was also carried out using a Tecnai G2 Spirit BioTWIN TEM (FEI company), operated at 80 kV. During sample observation, the number and the appearance of virions in each grid hole considered were recorded. A viral particle was classified as an intact virion if a continuous line could theoretically be drawn around its envelope. Conversely, if such a line could not be completed, the particle was categorized as a damaged virion.

### FASN activity assay

FASN enzyme activity was detected using a fatty acid synthetase activity assay kit (KTB2240; Abbkine). Briefly, 5 × 10^6^ cells were collected and lysed with 1 mL extraction buffer. Then, 20 μL sample, 16 μL NADPH, 4 μL Acetyl CoA, 8 μL Malonyl CoA, and 152 μL assay buffer were added to a 96-well plate, and the absorbance value at 340 nm was measured at 37 °C using a GloMax Discover Microplate Reader (Promega). The 10 s and 70 s absorbance values were recorded to calculate FASN enzyme activity.

### Lipidomics

Analysis was performed using two independent biological replicates (separate cell cultures) for each condition, with biological replicates analyzed in duplicate technical injections by LC-Q-TOF-MS resulting in a total 4 LC-MS measurements. Lipid extraction was conducted using a chloroform/methanol/water mixture acidified with formic acid. The procedure consisted of adding 600 µL of chloroform/methanol (1:2 v/v), 10 µL of formic acid, 5 µL of EquiSPLASH LIPIDOMIX (Avanti Polar Lipids), and glass beads to the sample. This mixture was vortexed for 20 s and then shaken for 5 min (2000 rpm, 4 °C). Subsequently, 200 µL of chloroform and 350 µL of deionized water were added, and the mixture was shaken again for 20 min at 2000 rpm and 4 °C. After centrifugation at 3900 x g for 10 min, the lower organic phase was collected and transferred to a chromatographic vial with a glass insert and diluted 10-fold with a 2:8 (v/v) water/methanol mixture. Extraction blanks without cells were also prepared as negative controls.

For lipidome analysis, an Agilent 1290 LC system equipped with a binary pump, online degasser, autosampler, and a thermostated column compartment, coupled to a 6540 Q-TOF–MS with a dual electrospray ionization (ESI) source (Agilent Technologies) was employed. Lipids were separated using a Kinetex EVO C18 reversed-phase column (2.1 × 150 mm, 1.7 μm particle size, Phenomenex) with a 0.2-μm in-line filter at 60°C. The mobile phase consisted of component A [5 mM ammonium formate in 20:80 (v/v) water/methanol] and component B [5 mM ammonium formate in 1:99 (v/v) water/methanol], pumped at a flow rate of 0.5 mL/min. The gradient elution started with 20% of component B, ramping to 100% from 0 to 15 min, and held at 100% B for 10 min. The column was then equilibrated with the starting conditions for 10 min. The total run time was 35 min, with an injection volume of 0.25 μL. Each extract was injected in duplicate. The SCAN acquisition mode recorded data in positive ion mode from 200 to 1700 m/z in high resolution (4 GHz). MS analysis parameters included a capillary voltage of 3500 V, fragmentation voltage of 120 V, nebulizing gas at 35 psig, and a drying gas temperature of 300°C. MS/MS analysis was performed with a collision energy set at 35 V and 80 V. The two most abundant peaks were selected for fragmentation and excluded for the next 0.3 min. MS/MS spectra were acquired in the m/z range of 50–1700. Lipid extracts were randomly injected with a quality control (QC) sample (pooled extracts) introduced after every 4 sample injections to monitor LC–MS system stability. Lipidomic data were processed using Agilent MassHunter Workstation Profinder 10.0 (Agilent Technologies). A list of identified lipids was created and used with the Targeted Molecular Feature Extraction algorithm. The extraction parameters were adjusted for positive ions, with charge carriers (H+ for PC, PC ether analogs, PE, PE ether analogs, SM) and a match of tolerance of 10 ppm, retention time window of 0.1 min, Gaussian smoothing applied before extracting ion chromatograms (EICs) based on a peak height threshold of 1000 counts. Peaks were manually inspected to detect false positive peaks. The .cef files were further processed in Mass Profiler Professional 15.1 software (Agilent Technologies) for data alignment and filtration, with missing values left as is. Alignment was configured with a slope of 0.0% an intercept of 0.1 min, a mass tolerance of 10.0 ppm, and an intercept of 2.0 mDa. Filtration criteria included the %RSD for lipid peak areas in QC samples and %RSD for internal standard peak areas in real samples, retaining molecular features (MFs) with a peak area %RSD below 25%.

Lipids were identified through a custom database search, using accurately measured m/z values within a 10 ppm tolerance. The identity of lipids and FA composition were verified manually using MS/MS spectra and Agilent Mass Hunter Workstation Lipid Annotator 1.0 software (Agilent Technologies). Identified lipid species were categorized by lipid class, total carbon atoms, and unsaturation level in fatty acyl groups, following LipidMaps classification standards (45). Diagnostic ions for lipid class identification included m/z 184.0726 for SM and PC identity, a neutral loss of 141.02 Da for PE, and m/z 264.27 for the C18 sphingoid base backbone.

### Statistical analysis

Statistical tests were performed using GraphPad Prism version 8.00 for Windows (GraphPad Software), Mass Profiler Professional 15.1 software (Agilent Technologies), or MetaboAnalyst5.0 (https://www.metaboanalyst.ca/home.xhtml). The CC_50_ (50% cytotoxic concentration) and IC_50_ (50% inhibitory concentration in plaque formation) values were calculated for each test using nonlinear regression (curve fitting analysis) in GraphPad Prism software. The selectivity index (SI) was determined as the ratio of CC_50_ to IC_50_. The data are presented as means and standard errors of the means (SEM). Differences were considered statistically significant when the *P*-value was < 0.05. Statistical details for each experiment including statistical significance and *n* value were provided in the figure legends.

## Supporting information

S1 Fig HSV-1–induced FASN upregulation occurs across multiple cell types. HFFs and HaCaT cells were infected with HSV-1 (MOI 1). **(A)** At 8, 24, and 32 hpi, total RNA was isolated and subjected to RT-qPCR to measure mRNA expression levels of FASN. The values were normalized to GAPDH and expressed as fold induction relative to mock-infected cells (set at 1) (*n* = 3; unpaired t-test). **(B)** Western blot analysis of protein lysates from mock or infected cells using antibodies against FASN, gD, or actin. A representative blot and the densitometric analysis are shown. Values were normalized to actin and plotted as fold induction relative to mock-infected cells (set at 1) (at least *n* = 2; unpaired t-test). **(C)** The FASN activity was examined in mock- and HSV-1-infected cells (*n *= 2; unpaired t-test). Data are shown as the mean ± SEM, *P < 0.05, **P < 0.01, ***P < 0.001.
(TIF)

S2 Fig FASN upregulation induced by HSV-1 is strain-independent. Western blot analysis of FASN, gD, and actin in SH-SY5Y cells infected with HSV-1 clinical isolates, MacIntyre, and 17 + (MOI 1) or left uninfected (mock) under serum-free conditions. Densitometric analyses and a representative blot are shown (*n* = 2; unpaired t-test). Data are shown as the mean ± SEM, *P < 0.05, **P < 0.01, ***P < 0.001.
(TIF)

S3 Fig HSV-1 increases *de novo* lipogenesis. The total amount of all examined lipid classes has increased in HSV-1 infected cells in comparison to uninfected cells and decreased in CMS121 or C75 treated HSV-1 infected cells in comparison to untreated HSV-1 infected cells. Total lipid content is the sum of all lipids belonging to a specific lipid class. Bars show mean ± SEM, *P < 0.05, **P < 0.01, ***P < 0.001 (unpaired t-test). Data are derived from at least two independent biological replicates (*n* = 2) analyzed in duplicate independent experiments for infected samples and uninfected control samples at the 48 hour time point. For the control uninfected condition at the 24 hour time point one biological replicate was used.
(TIF)

S4 Fig Cell viability in shCTRL and shFASN cells. **(A)** Representative images of shCTRL and shFASN cells at 48 h in serum-free medium. Cell viability was assessed using MTT assay **(B)** and crystal violet staining **(C)** (*n* = 3; unpaired t-test). Data are shown as the mean ± SEM, *P < 0.05, **P < 0.01, ***P < 0.001.
(TIF)

S5 Fig FASN modulation in the presence of serum. **(A)** SH-SY5Y cells transduced with lentivirus delivering short hairpin RNA (shRNA) targeting FASN (shFASN) or scramble RNA control (shCTRL) were analyzed by immunoblotting using antibodies against human FASN or actin, as a loading control, to confirm the efficiency of FASN protein depletion in presence of serum. A representative blot is reported (*n* = 3). **(B)** Western blot analysis of FASN and actin in shCTRL cells that were starved for 24 hours, followed by either serum replenishment or continued serum starvation until 48 h (*n* = 3).
(TIF)

S6 Fig Intact *vs.* damaged HSV-1 virions. Representative images showing HSV-1 intact **(A)** and damaged virions **(B)**.
(TIF)

S1 Table Primers for qPCR. (PDF)

S1 Data Raw data. (XLSX)
