# Supplementary figures and images for: The impact of fatty acid synthase on HSV-1 infection dynamics
Source: PLoS Pathog. 2025 May 6;21(5):e1013068. doi: 10.1371/journal.ppat.1013068 (PMC12084038; doi:10.1371/journal.ppat.1013068)

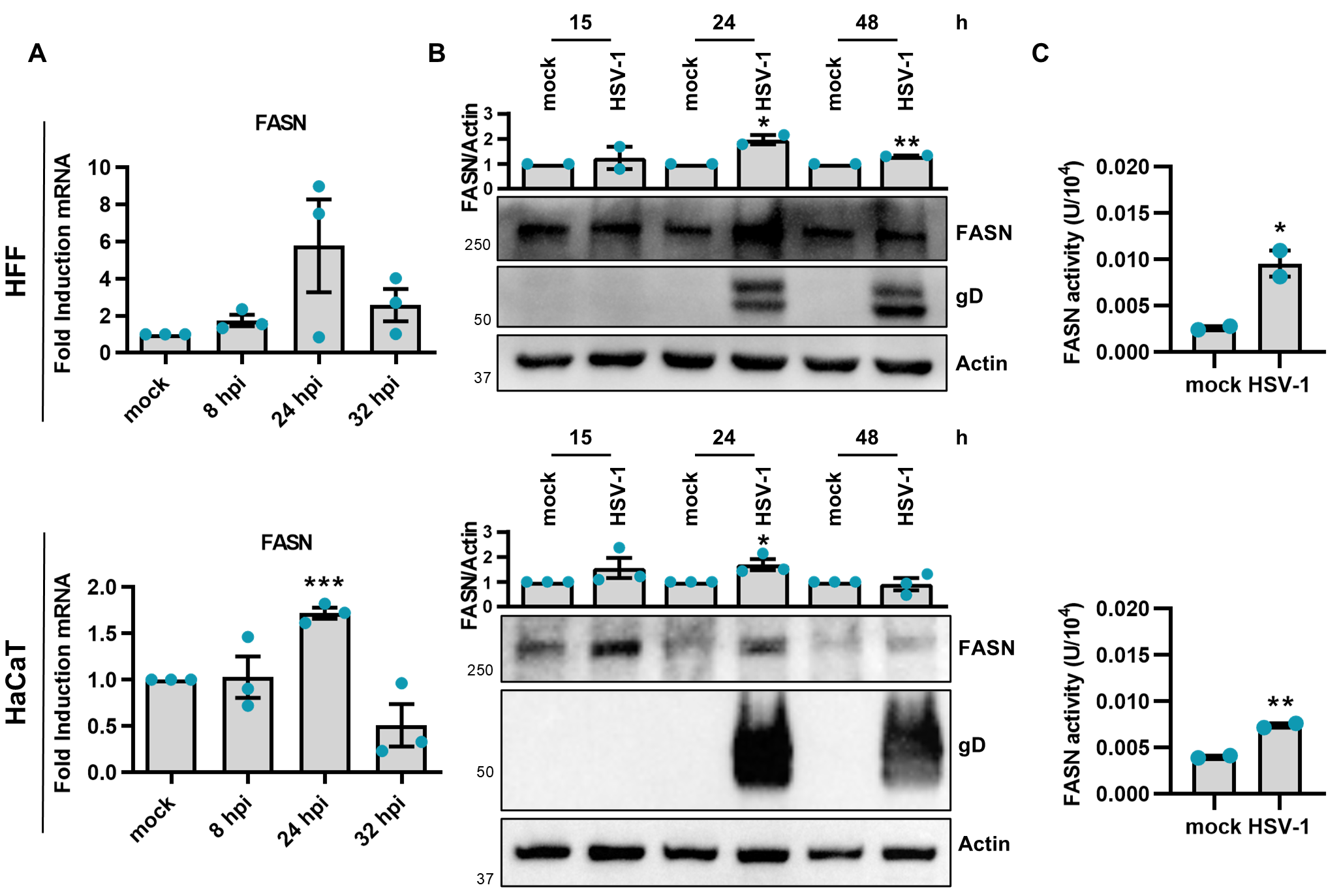

Supplement: S1 Fig — HFFs and HaCaT cells were infected with HSV-1 (MOI 1). (A) At 8, 24, and 32 hpi, total RNA was isolated and subjected to RT-qPCR to measure mRNA expression levels of FASN. The values were normalized to GAPDH and expressed as fold induction relative to mock-infected cells (set at 1) (n = 3; unpaired t-test). (B) Western blot analysis of protein lysates from mock or infected cells using antibodies against FASN, gD, or actin. A representative blot and the densitometric analysis are shown. Values were normalized to actin and plotted as fold induction relative to mock-infected cells (set at 1) (at least n = 2; unpaired t-test). (C) The FASN activity was examined in mock- and HSV-1-infected cells (n = 2; unpaired t-test). Data are shown as the mean ± SEM, *P < 0.05, **P < 0.01, ***P < 0.001. (TIF) [file ppat.1013068.s001.tif]

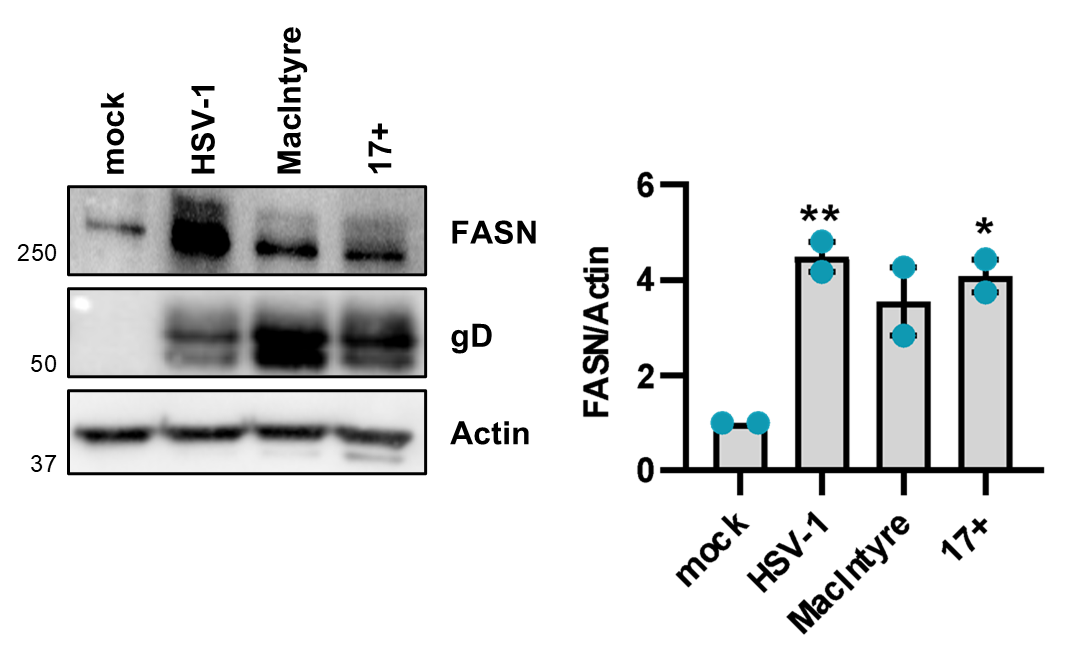

Supplement: S2 Fig — Western blot analysis of FASN, gD, and actin in SH-SY5Y cells infected with HSV-1 clinical isolates, MacIntyre, and 17 + (MOI 1) or left uninfected (mock) under serum-free conditions. Densitometric analyses and a representative blot are shown (n = 2; unpaired t-test). Data are shown as the mean ± SEM, *P < 0.05, **P < 0.01, ***P < 0.001. (TIF) [file ppat.1013068.s002.tif]

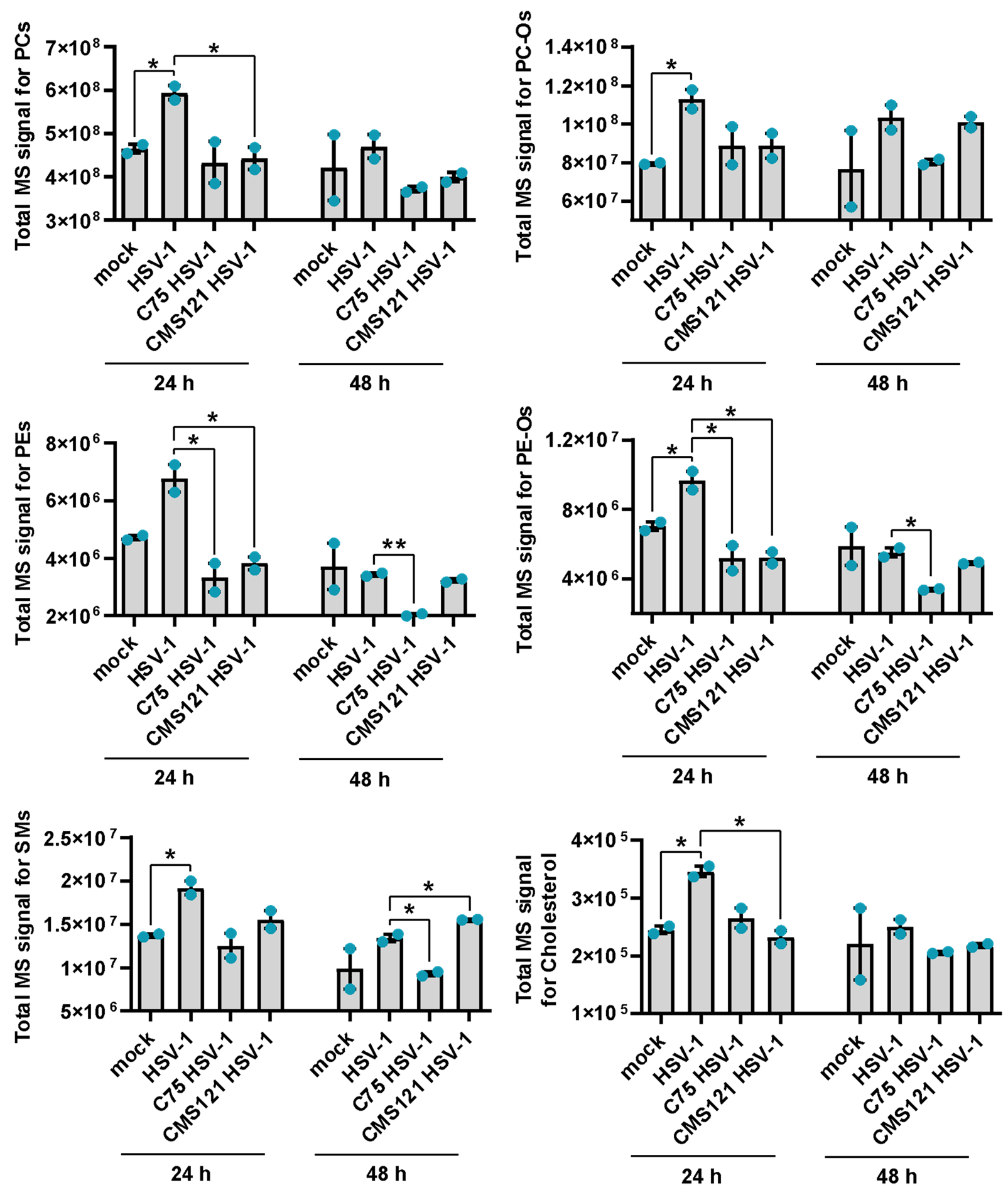

Supplement: S3 Fig — The total amount of all examined lipid classes has increased in HSV-1 infected cells in comparison to uninfected cells and decreased in CMS121 or C75 treated HSV-1 infected cells in comparison to untreated HSV-1 infected cells. Total lipid content is the sum of all lipids belonging to a specific lipid class. Bars show mean ± SEM, *P < 0.05, **P < 0.01, ***P < 0.001 (unpaired t-test). Data are derived from at least two independent biological replicates (n = 2) analyzed in duplicate independent experiments for infected samples and uninfected control samples at the 48 hour time point. For the control uninfected condition at the 24 hour time point one biological replicate was used. (TIF) [file ppat.1013068.s003.tif]

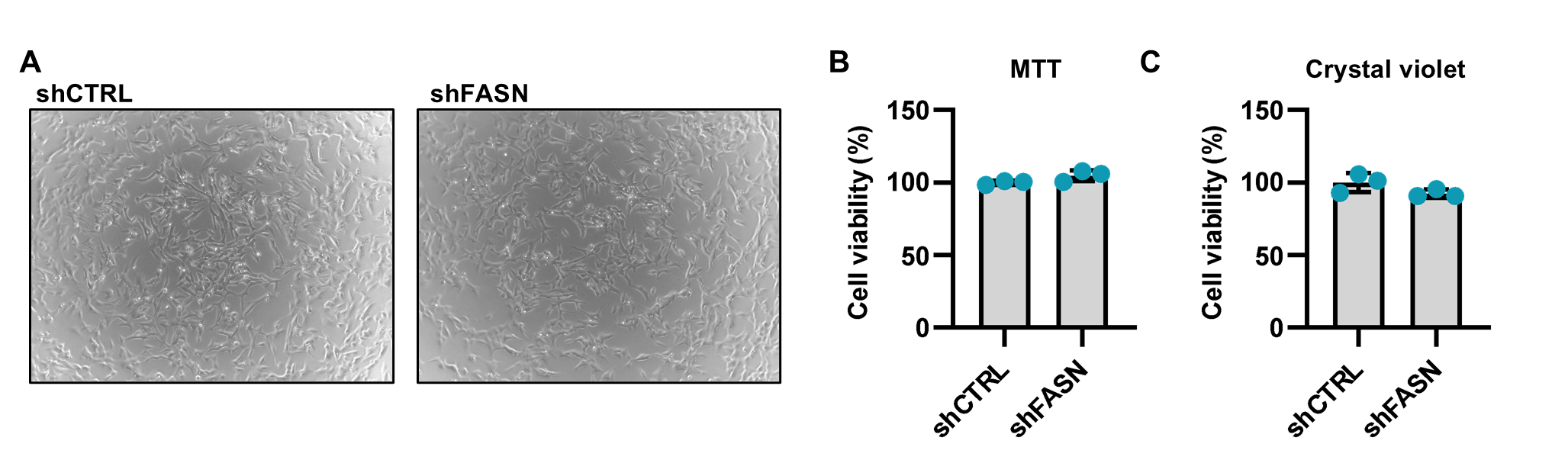

Supplement: S4 Fig — (A) Representative images of shCTRL and shFASN cells at 48 h in serum-free medium. Cell viability was assessed using MTT assay (B) and crystal violet staining (C) (n = 3; unpaired t-test). Data are shown as the mean ± SEM, *P < 0.05, **P < 0.01, ***P < 0.001. (TIF) [file ppat.1013068.s004.tif]

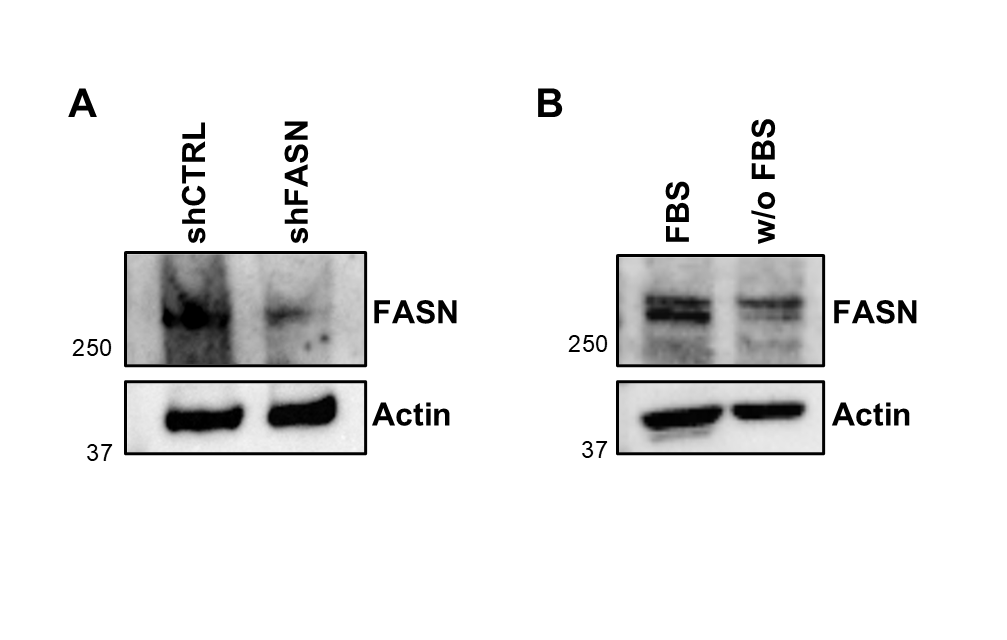

Supplement: S5 Fig — (A) SH-SY5Y cells transduced with lentivirus delivering short hairpin RNA (shRNA) targeting FASN (shFASN) or scramble RNA control (shCTRL) were analyzed by immunoblotting using antibodies against human FASN or actin, as a loading control, to confirm the efficiency of FASN protein depletion in presence of serum. A representative blot is reported (n = 3). (B) Western blot analysis of FASN and actin in shCTRL cells that were starved for 24 hours, followed by either serum replenishment or continued serum starvation until 48 h (n = 3). (TIF) [file ppat.1013068.s005.tif]

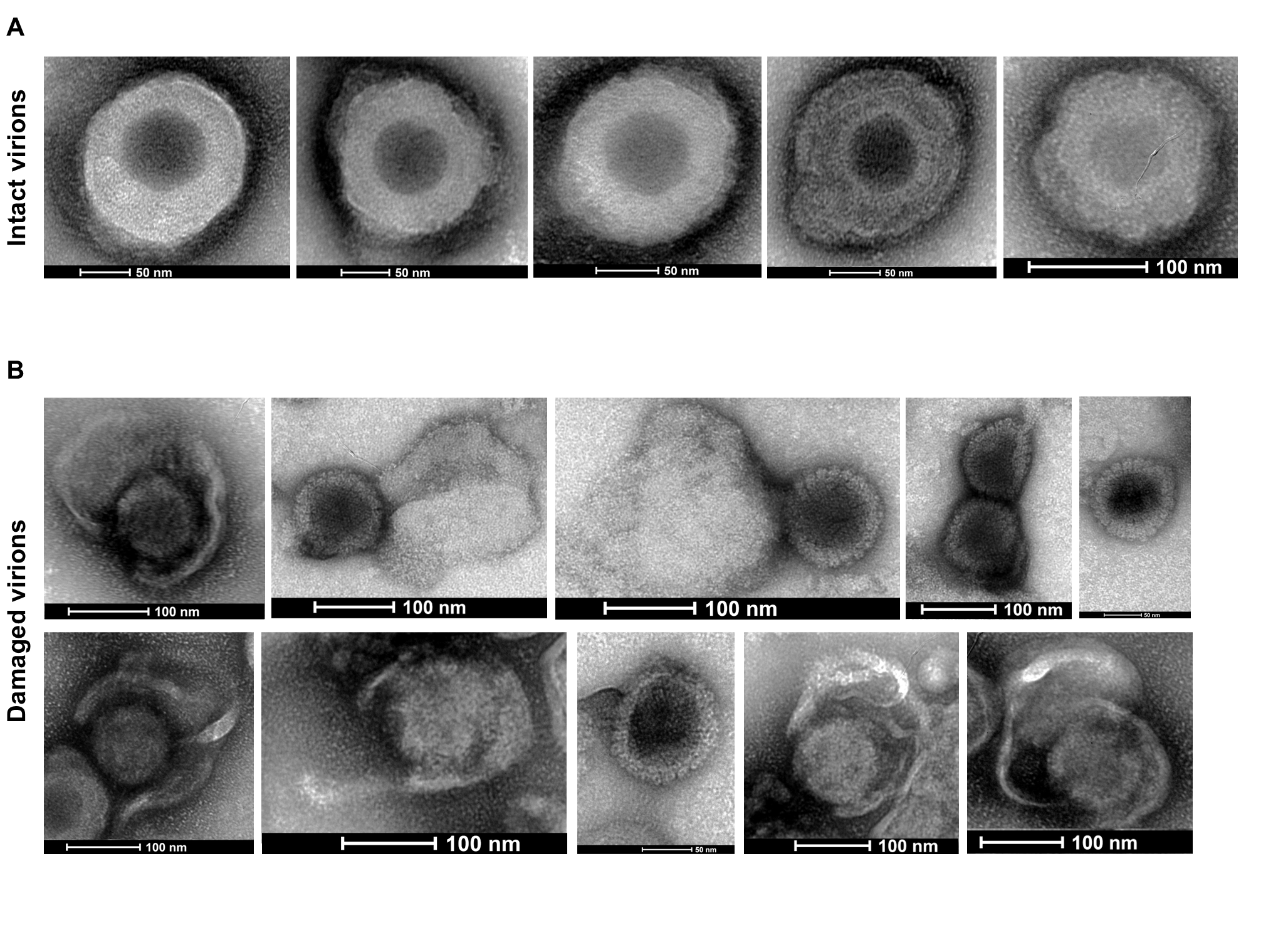

Supplement: S6 Fig — Representative images showing HSV-1 intact (A) and damaged virions (B). (TIF) [file ppat.1013068.s006.tif]
