# Supplementary material for: The impact of fatty acid synthase on HSV-1 infection dynamics
Source: PLoS Pathog. 2025 May 6;21(5):e1013068. doi: 10.1371/journal.ppat.1013068 (PMC12084038; doi:10.1371/journal.ppat.1013068)
Supplement: S1 Table — (PDF) [file ppat.1013068.s007.pdf]

S1 Table. Primers for qPCR.

Gene	Forward	Reverse
gE	5’-TGTCTGTATCAGCCGCAGC-3’	5’-TTCTGGAACACCCCGCGTA-3’
GAPDH	5’-AGTGGGTGTCGCTGTTGAAGT-3’	5’-AACGTGTCAGTGGTGGACCTG-3’
FASN	5’-AGGCTGAGACGGAGGCCATA-3’	5’-AAAGCTCAGCTCCTGGCGGT-3’
ICP0	5’-GGTCCCCACTGACTCATACG-3’	5’-ATCCCGACCCCTCTTCTTC-3’
Pol	5’-CGAGTGCGAAAAGACGTTCA-3’	5’-TGGAGGTGCGGTTGATAAAC-3’
